# The Measurement of Asthma and Allergic Rhinitis Control in Children and Adolescents

**DOI:** 10.3390/children7050043

**Published:** 2020-05-07

**Authors:** Maria Angela Tosca, Paolo Del Barba, Amelia Licari, Giorgio Ciprandi, Asthma and Rhinitis Control Study Group

**Affiliations:** 1 Pediatric Allergy Center, Istituto Giannina Gaslini, Genoa, 5-16146 Genova GE, Italy; mariangelatosca@gaslini.org; 2 Pediatric Unit, Università Vita-Salute San Raffaele, Milan, 58-20132 Milano MI, Italy; pauldelb@gmail.com; 3 Pediatric Clinic, Department of Pediatrics, Fondazione IRCCS Policlinico San Matteo, University of Pavia, Pavia, 65-27100 Pavia PV, Italy; a.licari@smatteo.pv.it; 4 Allergy Clinic, Casa di Cura Villa Montallegro, Genoa, 27-16145 Genova GE, Italy

**Keywords:** asthma, allergic rhinitis, control, children, adolescents

## Abstract

Asthma and allergic rhinitis (AR) are frequently associated. The objective of the treatment of asthma and AR should be the control of symptoms and disease progression. Therefore, the combined measurement of disease control is desirable. In this regard, a questionnaire able to together assess asthma and AR control has been validated: the CARAT (Control of Allergic Rhinitis and Asthma Test). A further pediatric version (CARATkids) has been generated. The current real-world study used different disease control measures in children and adolescents with asthma and rhinitis. A total of 138 children and adolescents were recruited at three allergy centers. CARAT, CARATkids, ACT (Asthma Control Test), cACT (children ACT), GINA (Global Initiative for Asthma) disease control classification, VAS (Visual Analog Scale) for asthma and nasal symptoms, and lung function were used in all subjects. There was a predominance of males (67.4%) and asthma was well-controlled (according to GINA classification) in about half the subjects. In children, the median CARAT and cACT values were 5 and 22 respectively. In adolescents, the median CARAT and ACT values were 23 for both tests. There were significant differences between CARAT and ACT (*p* = 0.035) as well as between CARATkids and cACT (*p* = 0.0001). However, the tests’ outcomes were different as assessed in different domains. CARAT and CARATkids are disease-control measurements that give additional information to other tests, therefore, these different questionnaires to measure disease control complement each other.

## 1. Introduction

It is well known that allergic rhinitis (AR) is a relevant risk factor for asthma exacerbations [1]. AR and asthma are closely associated both from a pathophysiological and a clinical point of view [2]. In this regard, a functional link has been described between nasal airflow and bronchial airflow [3] as well as a close association between upper and lower airway inflammation [4]. These concepts support the unbreakable relationship between the upper and lower airways, in health as well as in disease. As upper and lower airway diseases share the same pathophysiological features, their management and treatment should be integrated. In this regard, disease control is the shared goal for both AR and Asthma. Asthma control has been defined by the GINA (Global Initiative for Asthma) guidelines [5]. Consistently, AR control has been recognized [6]. Notably, AR comorbidity and consequent treatment represent a crucial step in severe and uncontrolled asthma [7].

The measurements of the asthma control can be performed by a grading proposed by GINA guidelines, specific questionnaires, including ACT (Asthma Control Test), or by the visual analog scale [8,9]. On the other hand, the measurement of AR control can be performed by VAS or specific questionnaires [10,11]. The idea of combining in one questionnaire the control measure of the two conditions had been proposed by a group of Portuguese doctors in 2007 [12]. The Control of Allergic Rhinitis and Asthma Test (CARAT) project was developed in a 3-phase pathway [12,13,14]. CARAT met nine of the 10 items on the COSMIN (Consensus-based Standards for the selection of health Measurement Instruments) checklist for evaluating the methodological quality of studies on measurement properties of patient-reported outcomes [15]. CARAT had adequate test–retest reliability, responsiveness, and longitudinal validity while confirming its high internal consistency and concurrent validity. Therefore, CARAT was proposed to be used both in clinical studies and in clinical practice, to compare groups and to evaluate individual patients over time. In this regard, CARAT was successfully used in primary care settings [16,17]. Moreover, it was validated in a variety of languages, including Brazilian and Dutch [18,19] and an electronic version [20]. It was also used in an Italian survey [21]. Further, a pediatric version has been developed and validated in children, the so-called CARATkids [22,23], and recently, in adolescents too [24]. Italian CARATkids has also been validated [25]. These studies concluded that CARAT can be used for the evaluation of AR and asthma in patients aged 14 years and onward; CARATkids and CARAT may both be used in adolescents. In clinical practice, there are various methods to measure the disease control, but it may be time-consuming to use questionnaires specific for asthma and AR, so to administer a single questionnaire could be advantageous.

Therefore, the current study evaluated a group of children and adolescents with AR and asthma in a real-world setting. The aim was to investigate the additional usefulness of CARAT/CARATkids in the management of children and adolescents with asthma and AR.

## 2. Materials and Methods

### 2.1. Patients

This cross-sectional study included a series of children and adolescents who were consecutively examined at 3 Italian Paediatric Allergy centers.

The inclusion criteria were: (i) age between 5 and 17 years; (ii) a documented asthma diagnosis, based on the history of intermittent wheezing, breathlessness, cough, and chest tightness in combination with reversibility to bronchodilators and/or positive response to bronchial methacholine challenge; and (iii) diagnosis of AR according to validated criteria [1]. In particular, allergy workup was based on the assessment of allergen-specific IgE (Immunoglobulin E), by performing a skin prick and/or serum assay; a positive test defined sensitization. An allergy was established if the exposure to the sensitizing allergen(s) induced symptoms. The exclusion criteria were: (i) the presence of asthma or AR alone; and (ii) concomitant diseases and treatments, including allergen immunotherapy, that may have affected the interpretation of the results.

The review ethics committees of the institutions involved approved the study procedure and written informed consent was obtained from all parents. Clinical data were recorded by an electronic case report form designed expressly for this study. The procedure was initially approved by the Ethics Committee of the Istituto Giannina Gaslini of Genoa (code number: 22253/2017; in the context of the Italian Project “ControL’Asma” promoted by the Italian Society of Paediatric Allergy and Immunology).

The visit included a careful history, mainly concerning asthma duration, current use of asthma medications including inhaled corticosteroids dosage (ICS) expressed as beclomethasone equivalence, oral corticosteroids use, rhinitis and allergy comorbidity, clinical examination, lung function testing (including bronchodilation testing), self-administration of the children Asthma Control Test (c-ACT) questionnaire or ACT for adolescents, asthma control level according to the GINA guidelines [5], and CARAT (for adolescents) and CARATkids (for children) questionnaires.

### 2.2. Functional Assessment

Spirometry was performed using a computer-assisted spirometer (Pulmolab 435-spiro 235, Morgan, England—predictive values ECCS 1993), with an optoelectronic whirl flow meter. This spirometer fulfills the ATS/ERS standards according to guidelines, and it was performed as stated by the European Respiratory Society [15,16]. In this study, the following parameters were used: forced expiratory volume in 1 s (FEV_1_) and forced expiratory flow at 25–75% of vital capacity (FEF_25-75_).

### 2.3. Asthma Control Level

Asthma control level was assessed according to the GINA criteria: patients were classified as having: well-controlled, partly-controlled or uncontrolled asthma [1].

### 2.4. Asthma Control Test

The ACT and cACT questionnaires consisted of 5 questions with 5 possible responses, exploring the patient’s perception of his/her asthma control [8]. In particular, cACT is implemented in clinical settings for children between the ages of four and eleven years; it is completed by the parents or by the children themselves [25].

The result could range between 0 and 25 or 27, where 25 or 27 is the optimal asthma control.

### 2.5. Visual Analog Scale (VAS)

The VAS consisted of one ruler asking for asthma symptoms perception [26]. In this study, the VAS was a 10-cm vertical line on which 0 implied the most severe respiratory symptoms, while 10 corresponded to no respiratory symptoms. Initially, patients were instructed to a mark on the line indicating their symptom perception at that moment. Thus, the lower was the numerical score marked by the patient, the greater was the perceived symptom severity. With a movable marker, the subject could mark any point on the 10-cm segment which best described his/her perception. No interval marker was visible on the line. A value >6 was considered good and suggested normal lung function [25]. VAS scoring was used to assess asthma and nasal symptoms. The last has been considered a surrogate marker for AR control assessment [27].

### 2.6. CARAT Questionnaires

The CARAT questionnaire is composed of 10 questions that address upper and lower airway symptoms, sleep interference, activity limitation, and the need to increase medication over 4 weeks. The answers are rated on a four-point scale, with a total possible score ranging from 0 (minimum control) to 30 (maximum control). The CARATkids has 17 questions, with two answer options: “Yes” scored as 1 = no control (symptom/item present) and “No” scored as 0 = control, such as the absence of symptoms or item not present. A part has to be completed by the child and another part by the parent. The sum of the scores was calculated.

### 2.7. Statistical Analysis

The distribution of each variable was checked using the Shapiro–Wilk W test. Descriptive statistics were performed and reported in terms of means with standard deviation (SD) or medians with inter-quartile ranges. For comparisons between two groups, the T-test for Student was used for normally distributed quantitative data. The ANOVA test was used to compare two or more groups of data by comparing the internal variability of these groups with the variability between the groups. The All-pairs test and the Tukey HSD test protected the significance of all combinations of pairs. The X square test was used to verify that the frequencies of the observed values adapt to the theoretical frequencies of a predetermined probability distribution. The relationship between variables was assessed using Spearman’s rank correlation coefficient. All tests were two-tailed and *p* values less than 0.05 have been considered as statistically significant. The program JMP Clinical 4.1 (SAS Institute, Milan, Italy) was used.

## 3. Results

This study included 138 patients, the mean age was 11.5 years, 93 were males and 45 females; there were 88 children and 50 adolescents. Demographic and clinical data are reported in Table 1.

**Figure 1:**
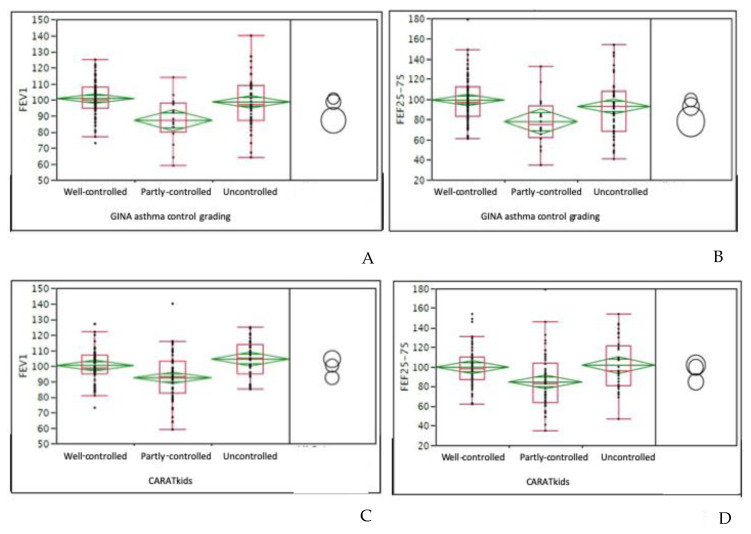
(**A**): associations between FEV_1_ (forced expiratory volume in 1 s) and GINA (Global Initiative for Asthma) asthma control grading in the whole sample; (**B**): associations between FEF_25–75_ (forced expiratory flow at 25–75% of vital capacity)and GINA asthma control grading in the whole sample; (**C**): associations between CARATkids (CARATkids: Control of Allergic Rhinitis and Asthma Test of pediatric version) grading and FEV_1_ in children; (**D**): associations between CARATkids grading and FEF_25-75_ in children. Data are expressed as medians and interquartile ranges. An ANOVA test was used. Circles concern the All-pairs Tukey-Kramer test.

In the total sample, made up of 138 subjects, according to the GINA grading, 78 (56.5%) patients had well-controlled asthma, 45 (32.6%) partly-controlled, and 15 (10.9%) uncontrolled. The mean VAS score was 7.2 and the median 8. Categorizing the VAS score, 116 (84%) patients had a good value, such as ≥ 6. The patients with uncontrolled asthma had the lowest FEV_1_ values (*p* = 0.0013) and FEF_25–75_ (*p* = 0.0083) in comparison with other patients (Figure 1A,B). The mean nasal symptom VAS was 6.9 (median 7); 107 (77.5%) had ≥ 6 scores, such as a good AR control. There was a significant association between good nasal VAS and good GINA asthma control (*p* = 0.01)

**Figure 2:**
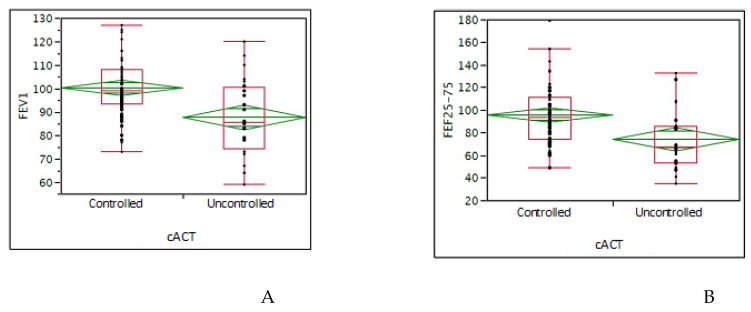
(**A**): comparison of FEV_1_ values between controlled and uncontrolled asthmatic children evaluated by cACT; (**B**): comparison of FEF_25-75_ values between controlled and uncontrolled asthmatic children evaluated by cACT (Asthma Control Test in children). Data are expressed as medians and interquartile ranges. The T-test was used.

In children, the mean cACT value was 21.8 (median 22); considering the cutoff ≥ 20: 64 (72.7%) had good asthma control, whereas 24 (27.3%) children had poor asthma control. The GINA asthma control grading showed that 48 (54.6%) children had well-controlled asthma, 29 (32.9%) partly-controlled, and 11 (12.5%) uncontrolled. A good VAS value was reported by 73 (83%) children. There was a significant association between cACT >20 and the highest FEV_1_ (*p* = 0.0001) and FEF_25-75_ (*p* = 0.0007) values (Figure 2A,B). There was also a significant association between GINA asthma control grading and FEV_1_ (*p* = 0.0084) values. There was a significant association between good nasal VAS and good GINA or cACT asthma control (*p* = 0.003 and 0.008, respectively).

In adolescents, the mean ACT value was 22.3 (median 23); considering the cutoff ≥ 20: 45 (90%) had good control, whereas 5 (10%) had poor control. The GINA asthma control grading showed that 30 (60%) adolescents had well-controlled asthma, 15 (30%) partly-controlled asthma, and 5 (10%) uncontrolled. A good VAS value was reported by 42 (84%) adolescents.

### CARAT Outcomes

**Figure 3:**
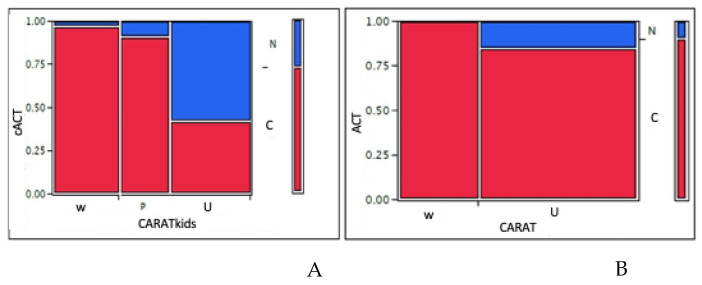
(**A**): comparison between cACT grades and CARATkids grades in children; (**B**): comparison between ACT grades and CARAT grades in adolescents. W = well-controlled asthma; P = partly-controlled; U = uncontrolled according to GINA classification. N = not controlled; C = controlled according with CARAT/CARATkids. A 1 value means 100% consistency between tests. X square test.

In children, the mean CARATkids value was 4.9 (median 5). Categorizing children: 30 (34%) children had a <3 score, such as good control of both asthma and AR, 22 (25%) children had a score between 4 and 5, such as a partial control, and 36 (41%) had poor control. Comparing CARATkids with GINA asthma control grading, the difference between asthma control groups was significant (*p* = 0.0001). Consistently, the comparison between cACT and CARATkids showed a significant difference among groups (*p* = 0.0001) as showed in Figure 3A. There was a significant association between CARATkids scores and FEV_1_ as well as FEF_25–75_ values (*p* = 0.026 and 0.016, respectively), as showed in Figure 1C,D. There was also a significant association between CARATkids and nasal VAS (*p* = 0.017).

In adolescents, the mean CARAT value was 22.5 (median 23). Categorizing the CARAT score: 17 (34%) adolescents had a ≥ 25 score, such as good control of both asthma and AR, whereas 33 (66%) adolescents had a score ≤ 24, such as poor control of both AR and asthma. Comparing CARAT with GINA asthma control grading, the difference between asthma control groups was significant (*p* = 0.0001). Consistently, the comparison between ACT and CARAT showed a significant difference among groups (*p* = 0.035) as showed in Figure 3B.

## 4. Discussion

Asthma and allergic rhinitis are frequently associated so that the term “united airway disease” has been coined and commonly used [28]. Currently, the control of asthma as well as of allergic rhinitis is considered the goal in the treatment of both diseases. However, the measurement of control may be performed using different tools, even though the outcomes are not always consistent [29,30]. For this reason, it is useful to consider more scoring in clinical practice. The attempt of having a unique questionnaire able to simultaneously grade both asthma and AR control has been pursued by a group of Portuguese doctors. This insight resulted in the preparation of the validated CARAT questionnaire [12,13,14]. It was used in some studies mainly performed in the primary care setting [16,17]. Further, a pediatric CARAT questionnaire was created (CARATkids) and adopted in very few studies [18,19,22,23]. However, CARATkids was never used in Italian children as well as CARAT in Italian adolescents. Therefore, the current study was performed to use both questionnaires in a real-world study enrolling both children and adolescents. This study evaluated different methods to assess asthma control, including GINA asthma grading, ACT, VAS, and CARAT.

At a glance, there was a clear predominance of asthmatic males as they made up 2/3 of the sample. The male predominance of asthma in childhood is a well-known phenomenon that conflicts with the female prevalence in adulthood and most likely depends on hormonal patterns that are age- and sex-related [31].

The asthma control grade was different considering the different scoring methods. Considering the GINA criteria, only 10% of subjects had uncontrolled asthma, whereas more than half had controlled asthma. Using the ACT questionnaire, more than 70% of children and 90% of adolescents had good asthma control. These discrepancies are acknowledged and depend on the different settings: GINA grading is based on reported symptoms and signs, whereas ACT considers the subjective perception of control. The AR control is eminently based on the perception of symptom severity, usually assessed by VAS. There was a significant association between nasal VAS scoring and CARATkids, but not in adolescents.

Therefore, the present study demonstrated that CARAT and CARATkids could be quite reliable tools in assessing asthma and AR control grades both in children and adolescents, even though some discrepancy emerged in comparison with other asthma and AR control measurements.

The preliminary studies, addressing the pediatric age, provided promising results that confirmed the adequate psychometric properties and reliability of CARATkids in childhood [22,23] and adolescence [24]. The Dutch validation study provided favorable outcomes concerning the reliability and validity of the test both in children and adolescents [18]. Consistently, the Brazilian study defined the cutoff to identify good or poor disease control [19]. Lastly, the study conducted only in adolescents confirmed the acceptable reliability of both tests in those subjects [24]. However, no real-world study tested CARAT and CARATkids in children and adolescents. The current study provided the first results in this setting. The findings are partially consistent with the literature data. On the other hand, the present outcomes could suggest that CARAT and CARATkids cannot exclude the assessment of other tests in asthma and AR control measurements.

The present study has some limitations including the cross-sectional design, a limited number of patients, the lack of biomarker evaluation, and CARATs were not validated in Italian. In this regard, a longitudinal study is ongoing, mainly concerning an evaluation of the association between treatments and disease control grading and enrolling a wider sample of patients. On the other hand, the strength of this study was the real-world approach. Real-world studies may provide information more adherent to daily practice than validation studies that involve selected patient populations which rarely mirror the real situation [32]. This real-life study provided interesting findings that may, therefore, faithfully mimic the daily medical activity of asthmatic pediatric outpatients. Moreover, these outcomes suggest that the use of CARAT/CARATkids could be useful in clinical practice. The possibility of using a questionnaire that together measures the control of the two diseases, might allow an easier approach in control assessment.

## 5. Conclusions

In conclusion, the current real-world study demonstrated that CARAT and CARATkids provide some interesting and complementary information about asthma and AR control grades. Therefore, the different questionnaires to measure disease control complement each other.

## Figures and Tables

**Table 1:** Demographic and clinical data in asthmatic children and adolescents.

	All Patients (N = 138)	Children (N = 88)	Adolescents (N = 50)
Age (mean, years)	11.5	9.9	14.3
Gender (male)	93/138 (67.4%)	58/88 (65.9%)	35/50 (70.0%)
GINA-based asthma control			
Well-controlled	56.5%	54.6%	60.0%
Partly-controlled	32.6%	32.9%	30.0%
Uncontrolled	10.9%	12.5%	10.0%
VAS Asthma (mean ± SD)	7.2 (±2.8)	7.0 (±2.8)	7.5 (±2.67)
FEV_1_ (mean ± SD)	98.0 (±13.7)	96.8 (±14.13)	101.5 (±12.75)
FEF_25-75_ (mean ± SD)	95.0 (±25.9)	89.8 (±27.2)	103.8 (±21.1)
VAS rhinitis (mean ± SD)	6.90 (±2.4)	6.96 (±2.4)	6.76 (±2.4)
cACT (mean ± SD)	N/A	21.8 (±3.86)	N/A
ACT score (mean ± SD)	N/A	N/A	22.3 (±2.66)
CARATKids (mean ± SD)	4.56 (±2.56)	4.90 (±2.53)	3.98 (±2.54)
CARAT (mean ± SD)	N/A	N/A	22.5 (±4.23)

VAS: Visual Analog Scale; FEV_1_: forced expiratory volume in 1 s; FEF_25–75_: forced expiratory flow at 25–75% of vital capacity; ACT: Asthma Control Test; cACT: children ACT; CARAT: Control of Allergic Rhinitis and Asthma Test; CARATkids: Control of Allergic Rhinitis and Asthma Test of pediatric version. N/A: not applicable.
